# Association of consumption of sugar-sweetened beverages with elevated blood pressure among college students in Yunnan Province, China

**DOI:** 10.1017/S1368980024000569

**Published:** 2024-02-29

**Authors:** Honglv Xu, Yun Zhao, Rui Tan, Min Li, Chunjie Yu, Danyun Rui, Jiangli Li, Yuan Xiong, Weibin Zheng

**Affiliations:** 1 School of Medicine, Kunming University, Kunming, Yunnan 650214, China; 2 Community Nursing Research Team of Kunming University, Kunming, Yunnan 650214, China; 3 Department of Infection Control, Yan’an Hospital of Kunming City, Kunming 650051, China; 4 The First People’s Hospital of Kunming, Kunming, Yunnan 650100, China; 5 Baoshan Center for Disease Control and Prevention, Baoshan, Yunnan 678100, China

**Keywords:** College students, Sugar-sweetened beverages, Hypertension, Prehypertension, Association

## Abstract

**Objective:**

Although some studies have examined the association between eating behaviour and elevated blood pressure (EBP) in adolescents, current data on the association between sugar-sweetened beverages (SSB) and EBP in adolescents in Yunnan Province, China, are lacking.

**Setting:**

Cluster sampling was used to survey freshmen at a college in Kunming, Yunnan Province, from November to December. Data on SSB consumption were collected using an FFQ measuring height, weight and blood pressure. A logistic regression model was used to analyse the association between SSB consumption and EBP, encompassing prehypertension and hypertension with sex-specific analyses.

**Participants:**

The analysis included 4781 college students.

**Results:**

Elevated systolic blood pressure (SBP) and diastolic blood pressure (DBP) were detected in 35·10 % (1678/4781) and 39·34 % (1881/4781) of patients, respectively. After adjusting for confounding variables, tea beverage consumption was associated with elevated SBP (OR = 1·24, 95 % CI: 1·03, 1·49, *P* = 0·024), and carbonated beverage (OR = 1·23, 95 % CI: 1·04, 1·45, *P* = 0·019) and milk beverage (OR = 0·81, 95 % CI: 0·69, 0·95, *P* = 0·010) consumption was associated with elevated DBP in college students. Moreover, fruit beverage (OR = 1·32, 95 % CI: 1·00, 1·75, *P* = 0·048) and milk beverage consumption (OR = 0·69, 95 % CI: 0·52, 0·93, *P* = 0·014) was associated with elevated DBP in males.

**Conclusion:**

Our findings indicated that fruit and milk beverage consumption was associated with elevated DBP in males, and no association was observed with EBP in females.

Hypertension is a global public health concern and a leading cause of death and disability in developing countries^(1)^. Hypertension refers to increased systolic blood pressure (SBP) and/or diastolic blood pressure (DBP) in a resting state (SBP ≥ 140 mmHg and/or DBP ≥ 90 mmHg)^(2)^. Prehypertension is characterised by blood pressure levels between normal and hypertensive ranges (120 mmHg ≤ SBP < 140 mmHg and/or 80 mmHg ≤ DBP < 90 mmHg). Additionally, the condition carries a risk and tendency to progress to hypertension^(2)^. Recent epidemiological studies have demonstrated that the incidence of hypertension in adolescents has significantly increased globally, especially in developing countries^(1)^. China Health and Nutrition Survey reports, according to the Chinese (2018), international (2016) and American (2017) diagnostic criteria for hypertension, the prevalence of hypertension in children and adolescents aged 7–17 increased from 4·4 % ∼ 8·9 % in 1991 to 12·8 % ∼20·5 % in 2015^(3)^. Moreover, hypertension in college students has received limited attention in recent years. Studies have suggested that the prevalence of hypertension among college students is > 30 %^(4,5)^. The prevalence of prehypertension among Chinese college students was 31·5 %^(6)^. Furthermore, the prevalence rate of hypertension among American college students was approximately 30·3 %, and of them, 90·7 % were not aware of their hypertension status^(5)^. In addition, a USA study suggested that 16·4 % of college students had elevated blood pressure (EBP)^(7)^. Essential hypertension, an influential risk factor for CVD in adolescents, is the main cause of hypertension^(8)^. Studies have discovered that common risk prediction models for hypertension have not yet been validated in adolescents^(1)^. Therefore, exploring the mechanisms underlying adolescent hypertension and investigating the aetiology of the condition are necessary.

Beverage consumption is increasing globally, and adolescents are the main consumers. A study on national beverage consumption in Brazil demonstrated that the consumption of whole and skimmed milk decreased from 2002 to 2018, while the consumption of other processed beverages increased^(9)^. A Saudi Arabian study reported that the average consumption of sugar-sweetened beverages (SSB), caffeinated beverages and carbonated beverages in college students was 4·2 l/week, 4·0 l/week and 1·5 l/week, 650·6 ml/d, 575·2 ml/d and 224·6 ml/d, respectively^(10)^. The consumption of SSB is a global health concern^(11)^. The number of deaths and burden of diseases attributed to SSB consumption have witnessed a significant increase in China between 1990 and 2019^(12)^. Brazilian researchers have examined measures to reduce SSB consumption and their positive health effects^(9)^.

Several studies have explored the association between SSB consumption and hypertension^(13)^. One study discovered that SSB intake was associated with hypertension in Norwegian women^(14)^. Multiple systematic reviews and meta-analyses have demonstrated that SSB consumption was a predictor of hypertension^(15–17)^. For instance, consumption of more than 250 ml/d of SSB was positively associated with hypertension risk (risk ratio (RR) = 1·07), with a linear dose–response^(15)^. Moreover, SSB intake (RR = 1·10) was positively associated with hypertension, while yoghurt (RR = 0·95) and pure fruit juice intake (RR = 0·97) were negatively associated with hypertension^(16)^. An increased SSB intake was associated with increased SBP in adolescents, and adolescents with excessive SSB were 1·36 times more likely to develop hypertension than those with low SSB consumption^(17)^.

Although a modest number of studies have explored the correlation between dietary behaviours and hypertension, evidence in adolescents is lacking. In particular, no reports are available on the association between dietary behaviour and hypertension in adolescents in Yunnan. Yunnan lies in Southwest China, which borders Myanmar, Vietnam and Laos. Influenced by climate and culture, Yunnan’s eating behaviour has its characteristics. The people of Yunnan like to eat baked, roasted and fried food and drink a variety of beverages. Currently, research on SSB consumption among students of Yunnan University is limited. In a preliminary survey of 528 students from Yunnan University, we discovered that the consumption rate of SSB was more than 20 %, which was higher than the consumption rate (17·5 %) of SSB among other college students (sample size was 585)^(18)^. A preliminary survey identified that SSB consumption was common among college students in Yunnan. Based on the results of the aforementioned reviews, this study aimed to explore the association between SSB consumption and EBP among college students in Yunnan Province, China. To verify this, we conducted a sampling survey among college students in Yunnan. The results provide clues to explore the etiological mechanisms of EBP and a basis for developing strategies to prevent EBP in college students.

## Methods

### Data source and participants

This study was part of a behaviour and sub-health study of Yunnan adolescents (BSSYA). This cross-sectional cohort study was designed to explore the effects of adolescent lifestyle on physical and mental health. The data used in this analysis were obtained from a cross-sectional survey. A questionnaire survey and physical examination were conducted between November and December 2021 among freshmen at Kunming University in Yunnan Province, China. A total of 6223 college students completed the questionnaire, with an effective rate of 100 %. Additionally, a total of 1422 participants who completed the questionnaire but did not participate in the physical examinations were excluded. Finally, 4781 participants were included in the analysis. Exclusion criteria: missing key variables in the questionnaire (e.g. SSB consumption), presence of logical errors in the questionnaire and missing blood pressure data. The average age of college students was (19·7 ± 1·6) years. Among them, 33·2 % (1586/4781) were male and 66·8 % (3195/4781) were female. Approximately, 73·9 % (3531/4781) Han and 26·1 % (1250/4781) ethnic minority groups were included in the analysis. The distribution was 79·2 % (3787/4781) in rural and 20·8 % (994/4781) in urban areas. The additional demographic variables are presented in Table 1.

**Table 1:** Distribution of elevated blood pressure in college students with different demographic variables

Variables	Categories	Elevated SBP				Elevated DBP			
		Yes	No	χ^2^	*P*	Yes	No	χ^2^	*P*
Sex	Male	971	615	711·15	< 0·001	915	671	334·83	< 0·001
	Female	707	2488			966	2229		
Major	Engineering	337	345	108·20	< 0·001	348	334	63·62	< 0·001
	Management	173	384			223	334		
	Pedagogy	258	575			288	545		
	Science	236	380			238	378		
	Agronomy	191	264			202	253		
	Literature	148	333			169	312		
	Medicine	161	366			195	332		
	Art	131	351			162	320		
	Other	43	105			56	92		
Age(year)	17–18	370	738	1·84	0·399	413	695	2·62	0·270
	19–20	859	1552			966	1445		
	≥ 21	449	813			502	760		
Nationality	Han	1222	2309	1·42	0·233	1368	2163	2·04	0·153
	Minority	456	794			513	737		
Residence	Rural	1342	2445	0·92	0·337	1503	2284	0·91	0·340
	City	336	658			378	616		
Only child	Yes	326	544	2·63	0·105	367	503	3·60	0·058
	No	1352	2559			1514	2397		
Family type	Two-parent family	1467	2659	5·21	0·157	1628	2498	1·11	0·776
	One-parent family	114	250			140	224		
	Combined family	70	155			84	141		
	Other	27	39			29	37		
Self-perceived socio-economic status	Worse	186	260	14·76	0·005	178	268	4·64	0·326
	Poor	404	728			446	686		
	Medium	994	1969			1157	1806		
	Good	72	120			74	118		
	Better	22	26			26	22		
Academic stress	Very heavy	108	164	7·11	0·130	115	157	6·44	0·168
	Heavy	573	1058			615	1016		
	General	960	1830			1108	1682		
	Easy	24	40			32	32		
	Very easy	13	11			11	13		
The number of close friends	0	25	35	14·17	0·003	24	36	3·80	0·284
	1–2	217	460			257	420		
	3–4	277	615			330	562		
	> 5	1159	1993			1270	1882		
Father’s education level	Illiteracy	332	581	1·49	0·828	366	547	2·01	0·734
	Elementary school	387	717			438	666		
	Secondary school	595	1123			654	1064		
	High school	236	425			269	392		
	University	128	257			154	231		
Mather’s education level	Illiteracy	546	987	1·74	0·784	614	919	1·81	0·770
	Elementary school	381	740			423	698		
	Secondary school	487	868			537	818		
	High school	166	310			192	284		
	University	98	198			115	181		
Father’s occupation	Civil servant	120	194	3·75	0·586	136	178	7·02	0·219
	Worker	58	93			61	90		
	Staff	89	170			96	163		
	Merchant	996	1839			1127	1708		
	Farmer	223	410			253	380		
	Other	192	397			208	381		
Mather’s occupation	Civil servant	76	160	1·05	0·958	89	147	2·96	0·706
	Worker	51	98			62	87		
	Staff	86	157			94	149		
	Merchant	1056	1947			1200	1803		
	Farmer	143	257			159	241		
	Other	266	484			277	473		
Family history of hypertension	Yes	22	46	0·23	0·633	23	45	0·88	0·348
	No	1656	3057			1858	2855		

SBP, systolic blood pressure; DBP, diastolic blood pressure.

Cluster sampling was used to include all college freshmen in the survey. After designing the questionnaire, peer experts reviewed and revised the questionnaire based on their opinions. A pre-survey was conducted among a small sample of college students to revise and improve the content of questions that participants could not comprehensively understand. The questionnaire, which was verified by experts and tested by pre-investigation, was produced as an electronic questionnaire using a questionnaire system (www.wjx.cn). During the questionnaire survey, college students gathered in the classroom. Trained investigators explained the purpose of the survey and highlighted guidelines for completing the questionnaire (including self-filling, completing the questionnaire independently, avoiding discussions with classmates and only filling in one questionnaire with one mobile phone). College students scanned the QR code of the electronic questionnaire using their mobile phones and completed the questionnaire, which took approximately 10–15 min. The investigators were asked to answer questions posed by the participants. Participants who completed the questionnaire volunteered to have their height, weight, chest circumference, vital capacity, blood pressure and other parameters measured. This was an anonymous survey, in which participants provided informed consent before participating. Participants were also informed that they could withdraw from the survey at any time. This study was approved by the Ethics Committee.

### Covariates

Covariates included socio-demographic and confounding variables. The socio-demographic variables assessed in this study included age, sex, only child status, residence, ethnicity, family type, parental occupation, parental educational background, number of close friends and self-evaluation of the family’s economic conditions. Confounding variables included smoking, drinking, BMI, academic pressure, family history of hypertension and academic pressure. Confounding variables reported in the literature may influence the blood pressure of college students. For instance, research has indicated that alcohol consumption was a significant predictor of hypertension^(19)^. The risk of hypertension in obese adolescents is four times higher than that in Italian adolescents of normal weight^(20)^. The National Health and Nutrition Examination Survey demonstrated that exposure to tobacco smoke was associated with hypertension in the USA^(21)^. Table 1 displays the categories of socio-demographic variables. The covariates were evaluated using a series of simple questions. The questions, for example, included, do you have a family history of hypertension (multiple family members with high blood pressure)? Two options were provided (yes or no): What kind of academic pressure do you think you have? The participants were presented with five options (very heavy, heavy, general, easy and very easy). How many times did you drink in the last month? Five options were provided (0, 1–2, 3–4, 5–6 and ≥ 7 times). 0 is not drinking, 1 or more is drinking. How many cigarettes have you smoked every day in the last month? Five options were available to choose from (0, 1–2, 3–4, 5–6 and ≥ 7 times). BMI was calculated by measuring the actual height and weight.

### Height, weight and blood pressure measurement

An ultrasonic height and Weight Meter (Beryl; BYH01BT) was used to measure the height and weight of the participants. The participants removed their shoes and coats and stood upright with their heels together. The heel, sacrum and scapulae were in contact with the column in a three-point-line standing position. Height and weight measurements were accurate to one decimal place. The BMI was calculated using height and weight data (BMI= weight (kg)/height (m)^2^). Four categories of BMI were included BMI: low weight (BMI < 18·5 kg/m^2^), normal weight (18·5 kg/m^2^ ≤ BMI < 24 kg/m^2^), overweight (24 kg/m^2^ ≤ BMI < 28 kg/m^2^) and obesity (BMI ≥ 28 kg/m^2^)^(22)^. An upper arm medical electronic sphygmomanometer (OMRON, J760) was used to measure blood pressure (mmHg), including SBP and DBP. Participants rested for 15 min before the measurement of their blood pressure. Blood pressure was measured using standard postures. Participants were seated, and their blood pressure was measured with a sphygmomanometer at the same level as the heart and right arm cuff. Participants diagnosed with high blood pressure had their blood pressure measured twice. Hypertension diagnostic criteria were recommended by the Chinese Guidelines for Hypertension Prevention and Treatment (2018 Revision)^(2)^: normal blood pressure (SBP < 120 mmHg and DBP < 80 mmHg), prehypertension (120 mmHg ≤ SBP < 140 mmHg and/or 80 mmHg≤ DBP < 90 mmHg) and hypertension (SBP ≥ 140 mmHg and/or DBP ≥ 90 mmHg). None of the participants underwent antihypertensive therapy before blood pressure measurements. Professional nurses measured blood pressure.

### Assessment of sugar-sweetened beverages

The dietary data of college students were collected through a dietary review survey. A semiquantitative FFQ developed by our research group was used to evaluate SSB consumption data^(23)^. The investigation focused on the eating behaviours of college students and the types of beverages available in the Chinese market, given the high consumption rate among college students. The questionnaire was developed after thorough discussions with the experts. SSB included carbonated, fruit and milk beverages, as well as tea and energy drinks. Carbonated beverages are drinks to which carbon dioxide gas is added under certain conditions and are mainly composed of sugar, colour, sweeteners and acid. Fruit beverages are not produced by simply squeezing fresh fruit without the addition of water or sugar. Instead, they are made by incorporating water, sugar, food colouring, preservatives and other additives. Tea beverages are prepared by steeping tea leaves in water and adding water, sugar or food additives. Energy drinks are special-purpose beverages with the main purpose of supplementing the energy required by the human body, and the main ingredients are water, vitamins, sugar, caffeine and other raw materials. Milk beverages are made by adding water, sugar and sweeteners to fresh milk or dairy products. The survey assessed the frequency of SSB consumption in the previous week. Five concise questions were asked to collect data regarding the five SSB. In the last 1 week, how many carbonated beverages did you consume per day (e.g. Coca-Cola, Sprite, 500 ml/bottle)? How many fruit beverages did you consume per day (e.g. orange, apple juice and 500 ml/bottle)? How many tea beverages did you consume per day (e.g. iced black tea, green tea and 500 ml/bottle)? How many energy beverages did you consume per day (e.g. Red Bulls, Hi Tigers and 500 ml/bottle)? How many milk beverages did you consume per day (e.g. Wang Zai milk, Yakult, 500 ml/bottle)? The participants were provided with four options for each question: 0 bottles, 1 bottle, 2–3 bottles and ≥ 4 bottles. The Cronbach’s α of semiquantitative FFQ in this survey was 0·92.

### Data analysis

The Excel database was exported to the questionnaire system for examination. Statistical analyses were performed using SPSS software (version 23.0; SPSS Inc.). Descriptive statistics, *χ* ^2^ tests and logistic regression models were also performed using the SPSS software. Descriptive statistics were used to calculate the proportions of the demographic variables, SSB consumption frequency and EBP prevalence. The *χ* ^2^ test was used to compare differences in the prevalence of EBP among college students with different demographic characteristics. A logistic regression model was used to analyse the association between SSB consumption and EBP. In this study, EBP included prehypertension and hypertension^(24)^. In the logistic regression model, the dependent variable, EBP, was binary (0 = normal blood pressure, 1 = EBP). Furthermore, SBP ≥ 120 mmHg was diagnosed as elevated SBP, and DBP ≥ 80 mmHg was diagnosed as elevated DBP. The independent variables consisted of five types of SSB consumption, each coded as binary (0 = no consumption, 1 = consumption). Two models were established in this study. Model 1 was not adjusted for variables, whereas model 2 was adjusted in the univariate analysis for statistically significant demographic variables, including smoking, alcohol consumption and BMI. OR were used to evaluate the strength of the association between SSB consumption and EBP. Statistical significance was set at *P* < 0·05.

## Results

Comparison of EBP detection rates among college students with different demographic variables

**Fig. 1:**
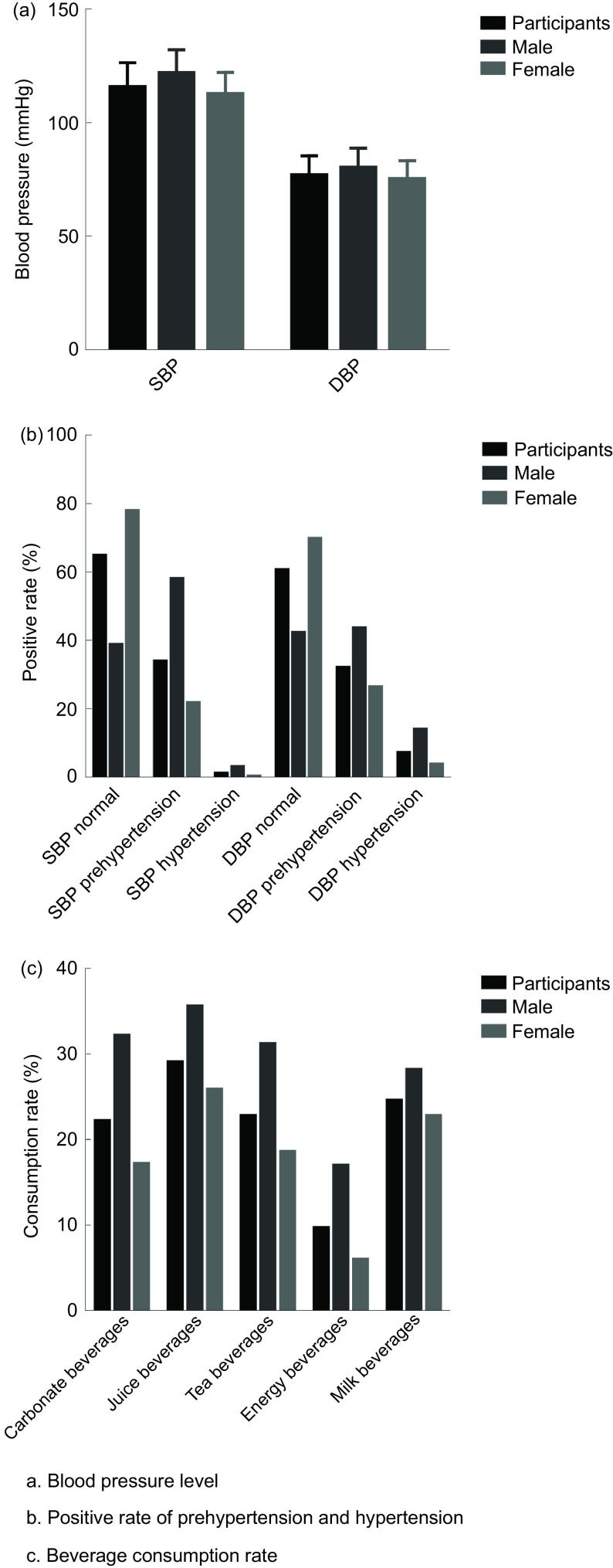
The level of hypertension, positive rate of hypertension and consumption rate of SSB in college students. SSB, sugar-sweetened beverages

The SBP level was (115·9 ± 10·5) mmHg, and the DBP level was (77·0 ± 8·4) mmHg in college students. The detection rates of elevated SBP and DBP were 35·10 % (1678/4781) and 39·34 % (1881/4781), respectively. Figure 1 displays the blood pressure levels and detection rates of EBP in college students of different sexes. Table 1 displays the distribution of EBP among college students according to different demographic variables. A statistically significant difference was observed in the detection rate of elevated SBP among college students with different sexes (χ^2^ = 711·15, *P* < 0·001), major (χ^2^ = 108·20, *P* < 0·001), self-evaluated family economic conditions (χ^2^ = 14·76, *P* = 0·005) and the number of friends (χ^2^ = 14·17), *P* = 0·003). Significant differences were also observed in the detection rate of elevated DBP among different sexes (χ^2^ = 334·83, *P* < 0·001) and majors (χ^2^ = 63·62, *P* < 0·001). No significant differences were identified in the detection rates of elevated SBP and DBP among the other demographic variables (*P* > 0·05).

### Association of sugar-sweetened beverages consumption with elevated blood pressure

**Fig. 2:**
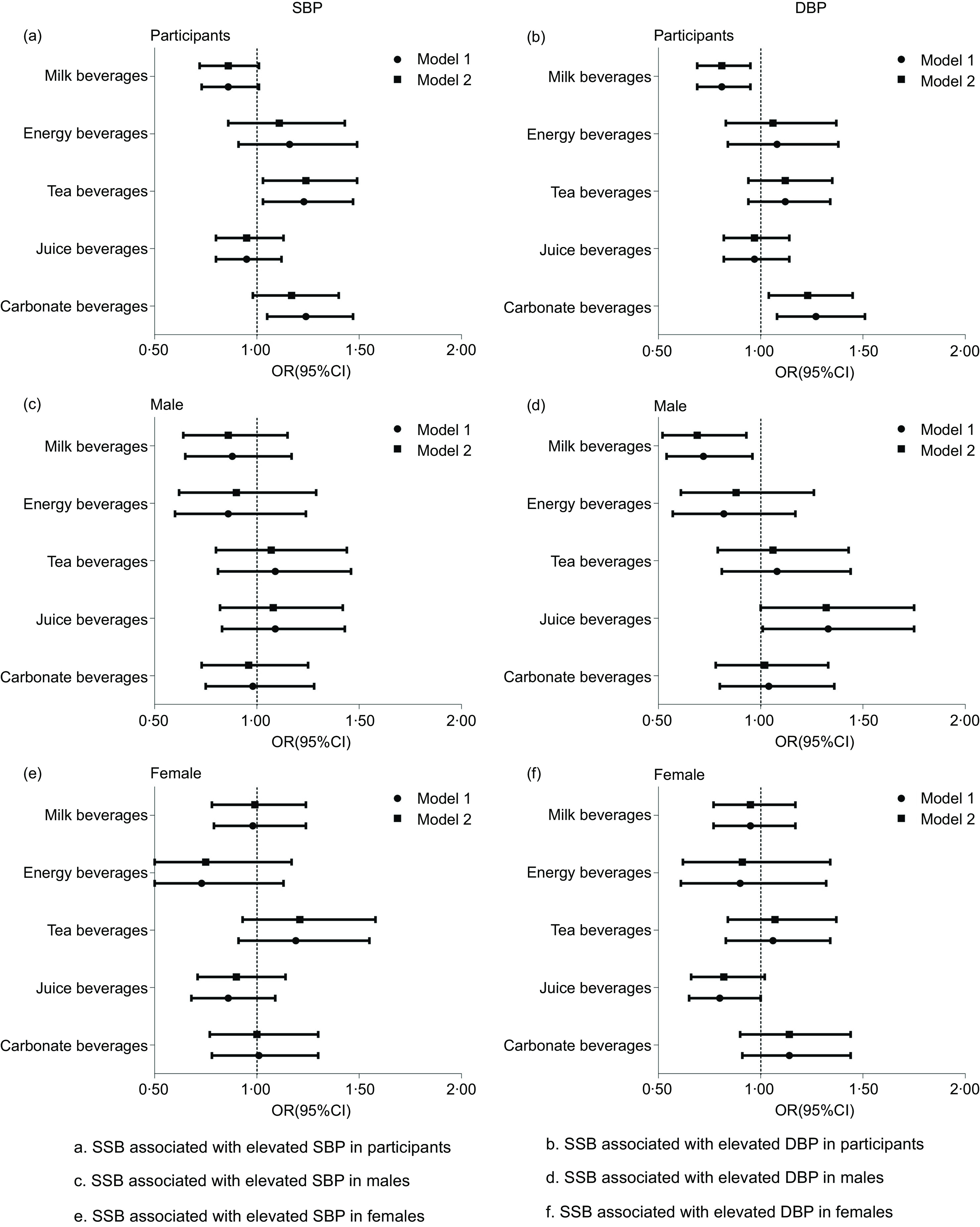
Association between SSB consumption and higher blood pressure in college students. SSB, sugar-sweetened beverages

The daily consumption rates of carbonated, fruit, tea, energy and milk beverages by college students in the recent 1 week were 22·2 % (1059/4781), 29·1 % (1392/4781), 22·8 % (1089/4781), 9·7 % (462/4781) and 24·6 % (1176/4781), respectively. The rates in males were 32·2 % (511/1586), 35·6 % (565/1586), 31·2 % (495/1586), 17·0 % (269/1586) and 28·2 % (447/1586), respectively. The rates in females were 17·2 % (548/3195), 25·9 % (827/3195), 18·6 % (594/3195), 6·0 % (193/3195) and 22·8 % (729/3195), respectively. Figure 1 displays the rates of SSB consumption among college students of both sexes. Table 2 displays the association between SSB consumption and elevated rates of SBP and DBP among college students. In logistic regression model 2 (adjusting variables such as major, self-evaluated family economic conditions, the number of friends, BMI, smoking and drinking), the analysis demonstrated that tea beverages (OR = 1·24, 95 % CI: 1·03, 1·49, *P* = 0·024) consumption was associated with an elevated SBP, and carbonated (OR = 1·23, 95 % CI: 1·04, 1·45, *P* = 0·019) and milk beverages (OR = 0·81, 95 % CI: 0·69, 0·95, *P* = 0·010) consumption was associated with an elevated DBP in college students. No association was observed between the consumption of other beverages and elevated SBP or DBP in college students (*P* > 0·05). In sex-specific analysis, fruit (OR = 1·32, 95 % CI: 1·00, 1·75, *P* = 0·048) and milk beverages (OR = 0·69, 95 % CI: 0·52, 0·93, *P* = 0·014) consumption was associated with elevated DBP in males. No association was observed between the consumption of other beverages and elevated SBP or DBP in males (*P* > 0·05). Additionally, no association was observed between SSB consumption and elevated SBP or DBP in females. (Fig. 2)

**Table 2:** Association between sugar-sweetened beverages consumption and elevated blood pressure in college students

Model	College student	Beverages	Elevated SBP						Elevated DBP					
			*β*	se	Wald	*P*	OR	95 %CI	*β*	se	Wald	*P*	OR	95 %CI
Model 1	Participants	Carbonate beverages	0·22	0·09	6·30	0·012	1·24	1·05, 1·47	0·24	0·09	8·12	0·004	1·27	1·08, 1·51
		Juice beverages	−0·06	0·08	0·43	0·511	0·95	0·80, 1·12	−0·03	0·08	0·15	0·697	0·97	0·82, 1·14
		Tea beverages	0·21	0·09	5·04	0·025	1·23	1·03, 1·47	0·12	0·09	1·68	0·196	1·12	0·94, 1·34
		Energy beverages	0·15	0·13	1·39	0·238	1·16	0·91, 1·49	0·08	0·13	0·38	0·539	1·08	0·84, 1·38
		Milk beverages	−0·15	0·08	3·33	0·068	0·86	0·73, 1·01	−0·21	0·08	6·36	0·012	0·81	0·69, 0·95
Model 1	Male	Carbonate beverages	−0·02	0·14	0·02	0·877	0·98	0·75, 1·28	0·04	0·13	0·09	0·762	1·04	0·80, 1·36
		Juice beverages	0·08	0·14	0·36	0·548	1·09	0·83, 1·43	0·28	0·14	4·13	0·042	1·33	1·01, 1·75
		Tea beverages	0·08	0·15	0·31	0·575	1·09	0·81, 1·46	0·08	0·15	0·26	0·609	1·08	0·81, 1·44
		Energy beverages	−0·15	0·18	0·64	0·424	0·86	0·60, 1·24	−0·20	0·18	1·21	0·270	0·82	0·57, 1·17
		Milk beverages	−0·13	0·15	0·79	0·374	0·88	0·65, 1·17	−0·33	0·15	5·08	0·024	0·72	0·54, 0·96
Model 1	Female	Carbonate beverages	0·01	0·13	0·00	0·967	1·01	0·78, 1·30	0·13	0·12	1·28	0·258	1·14	0·91, 1·44
		Juice beverages	−0·15	0·12	1·59	0·207	0·86	0·68, 1·09	−0·22	0·11	3·97	0·046	0·80	0·65, 1·00
		Tea beverages	0·17	0·13	1·68	0·195	1·19	0·91, 1·55	0·05	0·12	0·19	0·665	1·06	0·83, 1·34
		Energy beverages	−0·32	0·23	2·00	0·157	0·73	0·47, 1·13	−0·11	0·20	0·31	0·576	0·90	0·61, 1·32
		Milk beverages	−0·02	0·12	0·02	0·895	0·98	0·79, 1·24	−0·05	0·10	0·23	0·629	0·95	0·77, 1·17
Model 2	Participants	Carbonate beverages	0·16	0·09	3·18	0·075	1·17	0·98, 1·40	0·20	0·09	5·55	0·019	1·23	1·04, 1·45
		Juice beverages	−0·05	0·09	0·32	0·570	0·95	0·80, 1·13	−0·03	0·08	0·13	0·719	0·97	0·82, 1·14
		Tea beverages	0·21	0·10	5·10	0·024	1·24	1·03, 1·49	0·12	0·09	1·60	0·205	1·12	0·94, 1·35
		Energy beverages	0·10	0·13	0·59	0·442	1·11	0·86, 1·43	0·06	0·13	0·23	0·629	1·06	0·83, 1·37
		Milk beverages	−0·16	0·09	3·29	0·070	0·86	0·72, 1·01	−0·21	0·08	6·56	0·010	0·81	0·69, 0·95
Model 2	Male	Carbonate beverages	−0·05	0·14	0·11	0·744	0·96	0·73, 1·25	0·02	0·14	0·02	0·878	1·02	0·78, 1·33
		Juice beverages	0·07	0·14	0·27	0·601	1·08	0·82, 1·42	0·28	0·14	3·90	0·048	1·32	1·00, 1·75
		Tea beverages	0·07	0·15	0·21	0·650	1·07	0·80, 1·44	0·06	0·15	0·16	0·691	1·06	0·79, 1·43
		Energy beverages	−0·11	0·19	0·35	0·554	0·90	0·62, 1·29	−0·13	0·19	0·49	0·484	0·88	0·61, 1·26
		Milk beverages	−0·16	0·15	1·05	0·305	0·86	0·64, 1·15	−0·37	0·15	6·05	0·014	0·69	0·52, 0·93
Model 2	Female	Carbonate beverages	0·00	0·13	0·00	0·984	1·00	0·77, 1·30	0·13	0·12	1·24	0·266	1·14	0·90, 1·44
		Juice beverages	−0·11	0·12	0·78	0·378	0·90	0·71, 1·14	−0·20	0·11	3·22	0·073	0·82	0·66, 1·02
		Tea beverages	0·19	0·14	2·00	0·157	1·21	0·93, 1·58	0·07	0·12	0·31	0·580	1·07	0·84, 1·37
		Energy beverages	−0·29	0·23	1·62	0·203	0·75	0·48, 1·17	−0·10	0·20	0·23	0·631	0·91	0·62, 1·34
		Milk beverages	−0·02	0·12	0·02	0·901	0·99	0·78, 1·24	−0·05	0·11	0·27	0·607	0·95	0·77, 1·17

SBP, systolic blood pressure; DBP, diastolic blood pressure.

Model 1, unadjusted for variables.

Model 2, adjusted for major, the number of close friends, self-perceived socio-economic status, BMI, smoking and drinking.

## Discussion

The present study demonstrated that tea consumption was associated with elevated SBP, whereas carbonated beverage consumption was associated with elevated DBP in college students. Our results support the findings in different populations^(25,26)^. A cohort study reported that the highest quintile of SSB consumption was associated with an increased risk of hypertension in Norwegian females^(14)^. Results from a behavioural intervention trial demonstrated that reductions in SSB intake were significantly associated with reductions in SBP and DBP in adults in the USA^(27)^. Moreover, meta-analyses have suggested that high SSB consumption is associated with increased SBP in adolescents; participants with high SSB consumption are 1·36 times more likely to develop hypertension than those with low consumption of SSB^(16,17)^.

The results also revealed that fruit beverage consumption was positively associated with elevated DBP in males, and no association between SSB consumption and EBP was observed in females. Possible explanations include but are not limited to the following: First, males commonly have higher rates of hypertension than females^(28,29)^. Our data also confirm that males were twice as likely as females to have elevated blood glucose levels. Next, in comparison to females, males have a higher consumption of SSB^(30)^. In this study, the SSB consumption rate was significantly higher in males than in females.

Our data suggest that milk beverage consumption was negatively associated with elevated DBP in both college students and males. The negative association between dairy beverage consumption and elevated DBP may be related to the milk components of the beverages^(16,31,32)^. Studies have reported that dairy intake negatively correlates with the prevalence of hypertension^(33)^. American females aged 18–20 years who consumed at least two servings of dairy products per day had a 36 % reduction in risk of EBP^(34)^. Future research, especially cohort studies, is required to clarify the negative association between milk beverage consumption and elevated DBP.

The possible mechanisms of the association between SSB consumption and EBP are as follows. First, SSB constitutes a pro-inflammatory diet^(35)^. Pro-inflammatory diets may increase the risk of hypertension^(36)^. National Health and Nutrition Examination Survey demonstrated that pro-inflammatory dietary intake was associated with EBP in adolescents aged 12–18 years^(37)^. However, anti-inflammatory diets (e.g. fruits and vegetables) were associated with a reduction in blood pressure^(38)^. Second, SSB consumption affects sleep, and poor sleep quality is an influential factor for hypertension. Evidence already exists that adolescents who consumed carbonated beverages ≥ 3 times a day are 50 % more likely to report sleep disorders compared with adolescents who consumed carbonated beverages less than once a day^(39)^. Poor sleep quality is an independent risk factor for hypertension in Chinese youth. Furthermore, mild and moderate sleep disturbance increases the risk of hypertension^(40)^. Third, SSB consumption also increases the risk of obesity. Evidence exists that SSB consumption is significantly correlated with weight change^(41,42)^. In our study, obesity was an important predictor of hypertension^(43,44)^. Fourth, SSB consumption increases uric acid levels, which have previously been linked to blood pressure^(45)^. Experimental evidence strongly suggests that increased intracellular urate levels are a key factor in the pathogenesis of essential hypertension^(46)^. Fifth, SSB consumption exposes individuals to phthalic acid esters, which is associated with hypertension. Additionally, phthalic acid esters may be risk factors for hypertension^(47,48)^. For instance, di-(2-ethylhexyl)phthalate metabolite levels are associated with EBP in children and adolescents aged 6–19 years^(49)^.

The adverse health effects of SSB are a public health concern. Sweeteners in SSB are widely used in diets because of their low cost, and their consumption is increasing^(8,50)^. The results are consistent with our hypothesis and support the idea that SSB are consumed in excess and affect EBP in adolescents. Therefore, preventing and improving EBP by changing lifestyle, improving dietary behaviour and reducing SSB consumption may be of great significance^(51)^. As the prevalence of hypertension continues to rise in low- and middle-income countries, governments need to develop safe and effective policies to manage the condition, such as salt intake reduction, alcohol restrictions and tax increases for SSB^(52)^. A study reported that implementing a tax of 0·10 pounds per cup of SSB sold by a national chain of commercial restaurants in Britain led to a decline in the quantity of SSB sold by 11·0 % during the 12 weeks and 9·3 % at the 6 months^(53)^.

Our study had several strengths and limitations. The main strength is that this is the first study to explore the association between SSB consumption and EBP among college students in the southwest frontier region of China and fill a gap in related research. Another strength is that the study presented new findings, such as the negative association between milk beverage consumption and elevated DBP in college students. In addition, this study had a large sample size, and the study data provide a basis for the prevention and improvement of hypertension by changing the dietary behaviours of college students in Yunnan. Our study also had certain limitations. First, demographic variables and behavioural data were collected through questionnaires, which may have introduced information bias. Second, data on SSB consumption were collected using semiquantitative FFQ, which does not effectively assess intake. Third, the total energy intake may be a confounding factor in the association between SSB consumption and EBP. The total energy intake of college students was not adjusted for in this study, and we will further elucidate the impact of total energy intake on associations in a cohort study. Fourth, hypertension was measured only twice in participants with hypertension and only once in participants with normal blood pressure after 15 min of complete rest. Additionally, salt intake is difficult to assess given the characteristics such as liberalised eating behaviours in college students; therefore, the association analysis did not adjust for salt intake.

### Conclusion

Overall, we discovered that the detection rate of EBP (including prehypertension and hypertension) in college students in Yunnan was high. Additionally, SSB consumption was associated with EBP, and notable sex differences were present. These findings require further clarification in future cohort studies. More importantly, the government and schools are recommended to conduct comprehensive interventions on the eating behaviours of college students. This aims to motivate them to take more responsibility for their health, raise awareness of hypertension and implement preventive measures to reduce SSB consumption.
